# ZnO Nanomaterials and Ionic Zn Partition within Wastewater Sludge Investigated by Isotopic Labeling

**DOI:** 10.1002/gch2.202100091

**Published:** 2022-01-05

**Authors:** Miguel A. Gomez‐Gonzalez, Mark Rehkämper, Zexiang Han, Mary P. Ryan, Adam Laycock, Alexandra E. Porter

**Affiliations:** ^1^ Department of Materials and London Centre for Nanotechnology Imperial College London London SW7 2AZ UK; ^2^ Department of Earth Science & Engineering Imperial College London London SW7 2AZ UK; ^3^ UK Health Security Agency Centre for Radiation Chemical and Environmental Hazards Harwell Science and Innovation Campus Didcot OX11 0RQ UK

**Keywords:** isotopic labeling, ICP‐MS, primary sludge, wastewater treatment plants, ZnO nanomaterials

## Abstract

The increasing commercial use of engineered zinc oxide nanomaterials necessitates a thorough understanding of their behavior following their release into wastewater. Herein, the fates of zinc oxide nanoparticles (ZnO NPs) and ionic Zn in a real primary sludge collected from a municipal wastewater system are studied via stable isotope tracing at an environmentally relevant spiking concentration of 15.2 **µ**g g^−1^. Due to rapid dissolution, nanoparticulate ZnO does not impart particle‐specific effects, and the Zn ions from NP dissolution and ionic Zn display indistinguishable behavior as they partition equally between the solid, liquid, and ultrafiltrate phases of the sludge over a 4‐h incubation period. This work provides important constraints on the behavior of engineered ZnO nanomaterials in primary sludge—the first barrier in a wastewater treatment plant—at low, realistic concentrations. As the calculated solid–liquid partition coefficients are significantly lower than those reported in prior studies that employ unreasonably high spiking concentrations, this work highlights the importance of using low, environmentally relevant doses of engineered nanomaterials in experiments to obtain accurate risk assessments.

## Graphical abstract

The fate of ZnO nanomaterials in an untreated wastewater sludge at an environmentally relevant concentration is studied. Nanoparticulate ZnO dissolves rapidly within the acidic sludge. ZnO nanoparticles and ionic Zn show identical partitioning within the sludge. This suggests that the ecotoxicity of the ZnO nanomaterials at the µg g^−1^ level is entirely attributable to the dissolved Zn^2+^ ions.

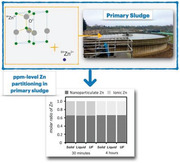

## 1 Introduction

Zinc oxide nanoparticles (ZnO NPs) have attracted a vast quantity of research owing to their wide range of optoelectronic and electrical properties, including transparency in the visible range, high infrared reflectivity, piezoelectric effects, and thermal stability.^[^ ^1^ ^]^ Commercially, ZnO NPs are utilized in cosmetics, paints, personal hygiene products, sunscreens, and moisturizers. They are also used as antibacterial agents in lotions, mouthwashes, and surface coatings to prevent microorganism growth.^[^ ^2^ ^]^ The worldwide production and use of ZnO nanoparticles continues to increase, rising from ≈550 t per annum in 2012 to an estimated 56 000 t in 2020, and this is associated with increasing release to the environment.^[^ ^3^, ^4^ ^]^ There thus exists a need to understand the behavior and transformation of ZnO NPs in real environmental milieu to assess their ecotoxicological impact.

One key pathway for release of engineered nanomaterials (ENMs) to the environment is through discharge from wastewater treatment plants (WWTPs). For such a scenario, it has been suggested that most released ZnO NPs will partition into the sludge—amounting to an estimated concentration of ≈24 µg g^−1^—that is subsequently amended to soils as biosolids or fertilizer.^[^ ^5^, ^6^ ^]^ Some of the particles (≈2.3 µg L^−1^) may also discharge into freshwaters as effluents. Nevertheless, the form of the Zn (i.e., whether it exists as particulate or ionic zinc) in the sludge and the effluent discharge in real environments remain to be elucidated, and such an understanding is needed to determine their toxicity and inform risk management. The fate of ZnO nanomaterials has been studied in synthetic, simulated, and real wastewater systems, mostly using X‐ray absorption spectroscopy.^[^ ^7^, ^8^, ^9^, ^10^, ^11^ ^]^ In these investigations, the spiking concentrations ranged from 10 to 1000 µg g^−1^ (Table S1, Supporting Information), and high doses of ZnO NPs are typically employed so that the speciation products can be determined within the detection limits of the employed techniques relative to the Zn background. These studies indicate that ZnO NPs in environmental media transform to several different phases, including ZnS and Zn_3_(PO_4_)_2_, and can associate with FeOOH under both acidic and neutral pH conditions. Such chemical transformations are important in limiting the bioavailability and toxicity of Zn^2+^ upon dissolution of the ZnO NPs.^[^ ^12^ ^]^ However, most previous studies were conducted at unrealistically high ZnO NP concentrations, and this may lead to nanomaterials aggregation and/or transformations into secondary phases. The fate, behavior, and chemical partitioning of ZnO NMs at µg g^−1^ concentration levels and in real environmental settings remain to be further investigated, with a particular paucity of data on the fate of ZnO NPs in primary sewage sludges.

Single particle—inductively coupled plasma mass spectrometry (ICP‐MS) is one of the few techniques that can detect low, environmentally relevant concentrations of ZnO NMs and subsequent dissolved species.^[^ ^13^ ^]^ One report showed that size exclusion chromatography (SEC) coupled with ICP‐MS can separate ZnO NPs from the released metal ions in environmental waters for quantification, with high recoveries (>97%) of the ions.^[^ ^14^ ^]^ An additional advantage of this technique is that it provides information about which compartment (i.e., liquid or solid matrix) of the sludge the particles and ions partition into, allowing prediction of their fate in the next stages of WWTPs, that is, if they discharge into treated effluent to the aquatic environment or are amended from sewage sludge to soil. Stable isotope labeling has emerged as an attractive alternative method that allows monitoring of trace amounts of elements that are highly variable in concentration within complex environmental systems.^[^ ^15^ ^]^ One key reason for the successful implementation of this approach in environmental and biological tracing is that the chemical properties of isotopically enriched materials do not deviate from their natural counterparts.

Herein, we study the distribution and partitioning of isotopically labeled ZnO NPs and a water‐soluble zinc salt (termed “ionic zinc” in the following) in untreated, acidic primary sludge collected from a WWTP based in UK (Anglian Water Ltd.), at an environmentally realistic concentration of 15.2 µg g^−1^. The primary sludge is of particular interest as it represents the first barrier that sewage waters encounter in their cleaning process. Prior investigations demonstrated that ZnO NPs in acidic sludges can undergo a range of physicochemical processes (including agglomeration and dissolution) in short periods of time, that is, within 3–4 h.^[^ ^9^, ^10^ ^]^ Therefore, the labeled ZnO NPs were spiked into the primary sludge and allowed to interact under stirring for 30 min and 4 h before the partitioning of the different Zn species between the compartments of the sludge was quantified using multiple collector ICP‐MS (MC‐ICP‐MS).

## 2 Results and Discussion

The employed ZnO NPs were isotopically enriched (to >99%) in ^68^Zn (^68^ZnO NPs) and synthesized via forced hydrolysis in diethylene glycol, as described elsewhere.^[^ ^16^, ^17^ ^]^ Furthermore, ionic zinc enriched in ^64^Zn (to >99%) was prepared as ^64^ZnCl_2_ by dissolving ^64^Zn metal in HCl and evaporation of the solution dryness.^[^ ^18^ ^]^ (see Supporting Information for details). To aid the discussion and clearly distinguish the added enriched ^64^Zn and ^68^Zn from the natural Zn (and its isotopes) present in the experimental system, the three distinct Zn species are abbreviated in the following as ^64^Zn‐en, ^68^Zn‐en, and Zn‐nat, as appropriate.

**Figure 1:**
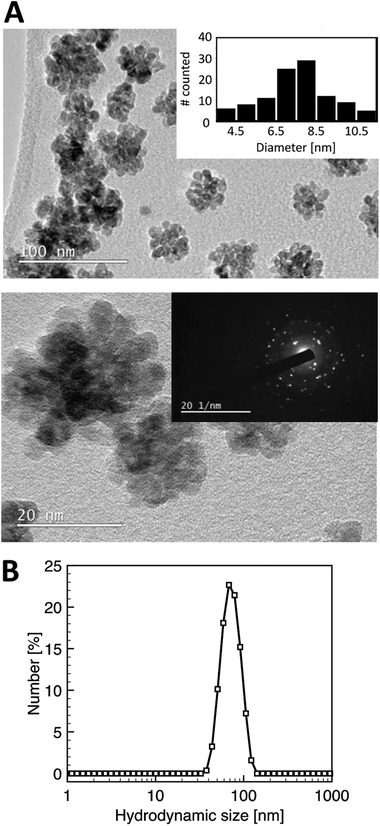
Characterization of isotopically labeled ^68^ZnO nanomaterials. A) TEM images of as‐synthesized ZnO NPs. Insets show the size distribution of individual NPs by TEM (sample size *n* = 105) and a representative selected‐area electron diffraction pattern of ZnO NPs. B) Hydrodynamic size distribution of ZnO particles as aggregates measured by dynamic light scattering.

Transmission electron microscopy (TEM) images show that the synthesized ^68^ZnO nanomaterials were mostly spherical in shape with an average diameter of 7.6 ± 1.7 nm (**Figure** **1**A). The deposited ^68^ZnO NPs existed as aggregates with apparent sizes varying from 10 to 50 nm under TEM. However, since the freshly synthesized ^68^ZnO NP suspension with a ^68^Zn‐en concentration of 680 mg L^−1^ was directly deposited onto the TEM grid for imaging, the observed agglomeration is likely a matter of the high concentration rather than indicative of the intrinsic tendency of the nanoparticle to aggregate. Their average hydrodynamic diameter is 71 ± 1 nm (Figure 1B), suggesting that the ^68^ZnO particles moved as small aggregates in their synthetic medium and possibly also in the sludge.

**Figure 2:**
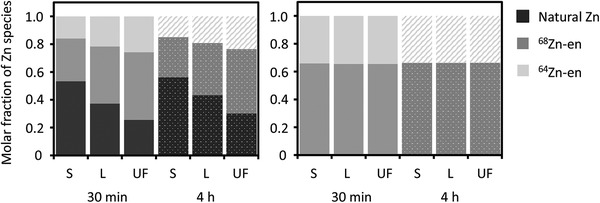
Partitioning of Zn species in sludges. Molar fractions of the different Zn species present in the samples, including natural Zn (left) and renormalized by excluding natural Zn contributions (right). Abbreviations: S = solid, L = liquid, UF = ultrafiltrate, en = enriched. The molar fractions have a bias and repeatability of better than 0.5%.

A total Zn‐en concentration of 15.2 µg g^−1^ from ^68^ZnO NPs and ^64^ZnCl_2_ was spiked into the untreated primary sludge. The ^68^Zn‐en and ^64^Zn‐en were thereby added at concentrations of 10.2 and 5.0 µg g^−1^, respectively, for a molar dosing ratio of 0.658: 0.342. Only a single dosing ratio is investigated since varying the dosing ratio from 0.5:0.5 to 0.93:0.07 does not appear to significantly impact the Zn partitioning behavior.^[^ ^19^ ^]^ To evaluate Zn partitioning within the sludge samples, three phases of interest are considered: solid sludge, liquid suspension, and ultrafiltrate (UF), where UF is a sub‐reservoir of the liquid suspension. The samples were allowed to equilibrate in polyethylene bottles (Figure S1, Supporting Information), and we compare ion exchange equilibria of the nanoparticulate ZnO and ionic zinc in the primary sludge at 30 min and 4 h, respectively, after spiking (**Table** **1** and **Figure** **2**).

**Table 1:** Molar fractions of isotopic Zn species in different samples analyzed by MC‐ICP‐MS

Sample^a)^	Molar fraction^b)^			Labeled molar fraction^c)^	
	Natural Zn f_Zn‐nat_	^68^Zn‐en f_68Zn‐en_	^64^Zn‐en f_64Zn‐en_	^68^Zn‐en f_68Zn‐en_′	^64^Zn‐en f_64Zn‐en_′
Solid (no Zn added)	1.00	0.00	0.00	–	–
Solid 30 min	0.53	0.31	0.16	0.66	0.34
Solid 4 h	0.56	0.29	0.15	0.67	0.33
Liquid (no Zn added)	1.00	0.00	0.00	–	–
Liquid 30 min	0.37	0.41	0.22	0.65	0.35
Liquid 4 h	0.44	0.38	0.19	0.67	0.33
UF (no Zn added)	1.00	0.00	0.00	–	–
UF 30 min	0.26	0.49	0.26	0.65	0.35
UF 4 h	0.30	0.46	0.23	0.67	0.33

^a)^ Descriptions for each sample can be found in Table S2, Supporting Information. Sample size *n* = 1

^b)^ for interpretation, the molar fraction of natural Zn, f_Zn‐nat_, refers to the relative molar quantity of natural Zn with respect to the total molar quantity comprising both natural Zn and the two enriched Zn species (^68^Zn‐en, ^64^Zn‐en). The measured concentrations of all three Zn species and their masses in the experimental system (comprising 10 g of sludge) are tabulated in Table S3, Supporting Information

^c)^ Renormalized molar fraction calculated only for the enriched Zn species ^64^Zn‐en and ^68^Zn‐en, excluding the natural Zn contribution. The molar fractions have a bias and repeatability of better than 0.5%.

At both time points investigated, the solid, liquid, and UF samples have different molar fractions of natural Zn relative to Zn‐en, whereby the Zn‐nat fraction is highest in the solid phase and lowest in the UF (Figure 2). The molar fraction of Zn‐nat also appears to increase slightly from 30 min to 4 h in all three phases. Closer scrutiny of the Zn mass balance (Figure S2, Supporting Information) reveals that only about 1/3 of the enriched Zn is accounted for in the analyzed samples, with the remaining likely adsorbed onto the plastic container walls, which act as an additional reservoir for Zn. A similar fate is likely for the natural Zn present in the experiments. This explains why the Zn mass budget was more variable than expected. The adsorption of transition metals such as Zn onto the surfaces of polyethylene containers is well‐documented in the literature, particularly at moderate to near‐neutral pH values.^[^ ^20^, ^21^ ^]^ Thirty min after spiking, an appreciable amount of Zn‐nat remained adsorbed to the container walls due to the initial increase in ionic strength upon spiking. From 30 min to 4 h, the system re‐equilibrated, and with slow desorption of Zn‐nat and (to a slightly lesser extent) Zn‐en from the container surfaces back to the solution phase (Figure S2, Supporting Information).

It is important to understand into which compartment and in what form ZnO NPs will partition in primary sludge under real conditions, for this dictates the mass transport of the NPs or their transformed species and thus their bioavailability in different phases of the sludge. A key observation in this context is that there is no analytically significant change in the molar ratio of ^68^Zn‐en (from the added ^68^ZnO NPs) to ^64^Zn‐en (from ^64^ZnCl_2_) between 30 min and 4 h for any of the three sludge phases. Furthermore, ^68^Zn‐en and ^64^Zn‐en are present in all three phases with an essentially identical ^68^Zn/^64^Zn ratio (Table 1, Figure 2). These findings demonstrate that the added ^68^ZnO NPs quickly dissolved to ionic ^68^Zn‐en within 30 min. The rapid dissolution of the ^68^ZnO NPs was thereby presumably aided by the slightly acidic pH of the sludge and the presence of organic humic acids at a concentration of 500 mg L^−1^. The latter conclusion is in accord with the results of previous investigations that studied the dissolution kinetics of ZnO NPs in acidic environments^[^ ^9^, ^10^ ^]^ and in the presence of natural organic matter (NOM).^[^ ^22^, ^23^ ^]^ Furthermore, the results show that the ionic ^68^Zn^2+^‐en from the dissolved ^68^ZnO NPs rapidly equilibrated with the ionic ^64^Zn^2+^‐en between all phases of the sludge. This demonstrates that no particle‐specific effects that stem from the initial presence of ^68^ZnO NPs are observed in the experiments. Rather, the ^68^Zn‐en and ^64^Zn‐en reveal identical partitioning, whereby they are likely adsorbed to the NOM of the sludge and present in free ionic form in the solid and the UF, respectively, and as both forms in the liquid phase. These findings are in agreement with the study of Laycock et al., who showed that ZnO NPs and ionic Zn behave indistinguishably in soil over a 12‐month incubation period, due to rapid dissolution of the ZnO NPs at acidic pH.^[^ ^19^ ^]^ Together with our data, this implies that any nanotoxicity observed at low ZnO NP concentrations should be attributed to dissolved Zn^2+^ ions, rather than the ZnO NMs in question—though the dissolution kinetics will be dependent on the nanomaterials.

To the best of our knowledge, there is only limited literature available on the partitioning of ZnO NMs in untreated primary sludge of WWTPs, let alone at low, and hence environmentally relevant, ZnO NP levels. The partitioning is generally characterized by a solid–liquid partition coefficient *D*_S/L_, which is defined as the concentration ratio of Zn species detected in the solid to that detected in the liquid phase. The experiments of this study thereby yielded *D*_S/L_ values for the ^68^Zn‐en (derived from the ^68^ZnO NPs) of 13 at 30 min and, as the system re‐equilibrates, of 9 at 4 h. Notably, analytically identical *D*_S/L_ values were obtained for the ^64^Zn‐en from water‐soluble ^64^ZnCl_2_ (Table S4, Supporting Information) and this corroborates the previous conclusion that the ^68^ZnO NPs rapidly dissolved in our experiments.

Using a concentration of ZnO NPs that was about 100× higher than the level employed in our work, Lombi et al. found that nearly all Zn stemming from the NPs partitioned into the solid phase of a mixture of primary and activated sludge. In detail, they determined *D*_S/L_ values of about 900 to 1600 for their systems, depending on the exact chemistry of the ZnO NPs.^[^ ^7^ ^]^ Most likely, the higher *D*_S/L_ values determined by Lombi et al. reflect formation of insoluble secondary Zn species from the ZnO NPs, and/or enhanced retention of dissolved Zn in the solid, for example by adsorption to NOM present, due to the use of high ZnO NP spiking concentrations. Regardless of the cause, the discrepant results of our study and those obtained in investigations that employed higher ZnO NP concentrations suggest that the use of environmentally unrealistic ZnO NP levels in partitioning studies for sewage sludge can produce findings that are only of limited relevance for understanding processes in actual WWTPs.

Chaüque et al. studied the fate of ZnO NPs in activated sewage sludge following addition of the particles at low nanoparticle concentrations of 5–20 µg g^−1^.^[^ ^24^ ^]^ In contrast to our study, Chaüque et al. found that nearly all Zn was retained in the activated sludge, with only an insignificant fraction of 1.3–2.1% (equivalent to *D*_S/L_ ≈ 47–76) detected in the effluent. The observation that the study of Chaüque et al. determined a higher *D*_S/L_ value when the sludge was spiked with a similar ZnO NP concentrations as in our study is of interest. Most likely, the discrepancy reflects the different compositions of the sludges that, in turn, are a consequence of the different processes and conditions by which they are produced. In detail, Chaüque et al. studied a pH‐neutral activated sludge whilst an acidic primary sludge was investigated in the current study. Indeed, the type of sewage sludge can impact the partitioning behavior, which has been observed for other chemical compounds such as pharmaceuticals ingredients.^[^ ^25^ ^]^

Lastly, the environmental implications may be different from those previously alluded to. At a nanoparticle dosing concentration as low as 10.2 µg g^−1^, we expect no transformation of ZnO nanomaterials into secondary phases via dissolution–reprecipitation in primary sludges. This is because i) there is a much reduced chemical driving force for precipitation compared to high concentrations of ZnO, and ii) the presence of NOM in sludges can limit the bioavailability of Zn^2+^ ions through complexation, where Zn^2+^ preferentially binds to the amine and carboxylic moieties in NOMs.^[^ ^26^ ^]^ NOMs, dissolved organic matter, and exopolymeric substances can also directly adsorb onto nanoparticulate ZnO, forming dynamically evolving eco‐corona and altering their dissolution characteristics.^[^ ^27^ ^]^ Therefore, without chemical speciation, detoxification mechanisms such as sulfidation may be of little relevance at environmentally low ZnO NP concentrations. Further high‐sensitivity in situ chemical imaging studies on a wide range of nanomaterial concentrations (from a few to hundreds of µg g^−1^) are needed to corroborate this. Moreover, the low solid–liquid partition coefficients *D*_S/L_ calculated in this work imply that the partitioning of ZnO NPs in the form of dissolved Zn^2+^ ions to the liquid phase of the sludge may have been underestimated. Since ionic zinc is more mobile compared to its particulate counterparts, it can easily leach into effluent waters from the primary sludge. Complexation and chelation of metals ions have been exploited by nature to transport micronutrients to plants.^[^ ^26^ ^]^ By the same mechanism, Zn^2+^ that is partitioned to the nonsolid phase of the sludge may lead to an increase in their concentration in effluent waters. Nanotoxicity of particulate ZnO has been mostly attributed to their dissolution to form Zn^2+^ ions, and Zn^2+^ ions above a threshold dose are known to impart high toxicity toward microorganisms in wastewater solutions^[^ ^28^, ^29^ ^]^ and plants in amended soils.^[^ ^30^, ^31^ ^]^ In a recent study by Lee et al*.*, the authors show that the presence of NOM reduces the toxicity of ZnO NPs in terms of algal‐growth inhibition, where the reported EC_50_ concentrations are on the order of 100s of µg g^−1^.^[^ ^32^ ^]^ For 10 µg g^−1^ or less, there is a negligible difference in the growth inhibition with and without the presence of NOM. In the same study, decreased toxicity was reported in a *Daphnia magna* immobilization test for µg g^−1^ level of ZnO NPs due to the presence of NOM. These data imply that the in situ toxicity of ZnO NPs at low concentrations in effluents are microorganism‐dependent, and so is the effect of NOM complexation on ZnO ENM ecotoxicity. The adverse effects of dissolved and/or complexed Zn^2+^ ions at µg g^−1^ concentrations on subsequent stages of the wastewater treatment process, such as on the activity of activated sludge or anaerobic digestion, have been documented elsewhere.^[^ ^33^, ^34^ ^]^ Concerning the solid phase of the untreated primary sludge, the Zn^2+^ ion concentration in the solid sludge that becomes treated and amended to soil will be lower at environmentally relevant concentrations. Hence, the imparted toxicity should be low at µg g^−1^ concentrations, and Zn remediation in primary sludge treatment needs no special attention.

## 3 Conclusions

In conclusion, the fate of ZnO NPs and ionic zinc from ZnCl_2_ in the primary sludge of a WWTP were investigated at an environmentally relevant dosage of 15.2 µg g^−1^ using stable isotope labeling and analyses by MC‐ICP‐MS. Monitoring of two Zn species revealed that Zn from the ZnO NPs distributed in exactly the same manner as the ionic Zn derived from the ZnCl_2_ in the solid, liquid, and ultrafiltrate phases of the sludge within 30 min. This demonstrates that at the investigated concentration, the ZnO NPs dissolved rapidly to release Zn^2+^ ions. As a consequence, the experiments revealed no specific nanoparticulate effects from the initial addition of ZnO NPs. The analyses furthermore yielded solid–liquid partition coefficients for Zn derived from the ZnO NPs of 13 and 9, at 30 min and 4 h post‐spiking, respectively. These partition coefficients are significantly lower compared to values obtained in similar experiments that were conducted at higher, but environmentally unrealistic, ZnO NPs levels. In addition, our values differ from those reported for activated sludge at similarly realistic dosing, which implies that wastewater and compositions also affect the fate of released engineered nanomaterials. Overall, the data presented here suggest that Zn^2+^ toxicity is of more importance than previously thought at low concentrations of ZnO NPs in primary sewage sludge, as highly mobile Zn^2+^ ions can easily leach into the effluents.

## 4 Experimental Section

### Synthesis and Characterization of Isotopically Labeled Zn Species

Synthesis of the isotopically labeled Zn species and all subsequent sample preparation work was carried out in the clean room facilities of the MAGIC Laboratories at Imperial College London using distilled mineral acids and Milli‐Q water of ≥18.2 MΩ cm quality (Millipore, UK). Zinc that was artificially enriched in the isotopes ^64^Zn and ^68^Zn to 99.7% and 99.1%, respectively, was purchased as metal powders from Isoflex USA. The synthesis of isotopically labeled ^68^ZnO nanoparticles from the ^68^Zn‐en was via forced hydrolysis in diethylene glycol, as described elsewhere.^[^ ^16^, ^17^ ^]^ Briefly, a ^68^Zn acetate precursor was first prepared from the ^68^Zn metal. The ^68^Zn acetate was dissolved in diethylene glycol and gently heated before water was added to force hydrolysis and precipitation of the ^68^ZnO NPs. This produced a suspension of ^68^ZnO NPs in diethylene glycol with a ^68^Zn‐en concentration of 680 mg L^−1^. Soluble, isotopically labeled ^64^ZnCl_2_ was prepared by dissolving ^64^Zn metal in 6 m HCl.^[^ ^18^ ^]^ This solution was evaporated to produce ^64^ZnCl_2_, which was subsequently dissolved in Milli‐Q water to a ^64^Zn concentration of 75 mg L^−1^.

The particle size, shape, and crystal structure of the ^68^ZnO NPs were determined using TEM and electron diffraction, respectively. For TEM imaging, a freshly synthesized batch of ^68^ZnO NPs was deposited directly onto holey carbon support copper grids (300 µm mesh, *TAAB*), dried for 12 h in a desiccator, and subsequently imaged with a JEOL STEM/TEM 2100Plus microscope. Particle size distribution was determined from the collected TEM images using ImageJ. Dynamic light scattering was also performed on the ^68^ZnO NP suspension with a Malvern Zetasizer Nano ZS. Three measurements were undertaken with the average reported.

### Incubation of Labeled Zn Species in Wastewater Sludge

The primary sludge was collected from a typical municipal WWTP in the South East of England (Anglian Water Group Ltd.) and stored in glass containers. A previous publication by the group reports the chemistry of this sludge and can be referred to in ref. [10]. These include: pH = 5.2, total organic carbon = 336 mg C L^−1^ and water content of the sludge = 95.88% by mass.

For the experiments, acid‐cleaned polyethylene (PE) bottles were filled with 10 g samples of primary wastewater sludge. Within 30 min, two of the three experiments were treated with a total of 152 µg of ^64^Zn‐en and ^68^Zn‐en, for a total Zn‐en concentration of 15.2 µg g^−1^ (Tables S2 and S3, Supporting Information). For the untreated experiment, the ≈10 g of untreated primary sludge was sampled at this time and found to have a Zn‐nat concentration of 7.88 µg g^−1^, for a total Zn‐nat content of ≈79 µg (Tables S2 and S3, Supporting Information). In detail, each treated experiment was spiked with 102 µg ^68^Zn‐en in the form of ^68^ZnO NPs and 50 µg ^64^Zn‐en in the form of dissolved ^64^ZnCl_2_, for a molar ratio of ^64^Zn: ^68^Zn of 0.342: 0.658 when isotopic enrichment and mass differences were considered. This was achieved as follows. From the ^68^ZnO NP stock suspension, 150 µL were pipetted to each sludge sample for a ^68^Zn‐en concentration of 10.2 µg g^−1^. Similarly, 666 µL of the ^64^ZnCl_2_ stock solution were added to each sludge sample, for a final ^64^Zn‐en concentration of 5.0 µg g^−1^. Upon spiking, the PE bottles with the sludge were shaken to achieve a homogeneous distribution of the labeled Zn species and left to incubate for either 30 min or 4 h.

To evaluate Zn partitioning within sludge samples, three phases of interest were considered: solid sludge, liquid suspension, and an ultrafiltrate (UF) fraction, which was the dissolved fraction with particles of less than about 2–3 nm in size. These were obtained as follows. Three filtration devices were prepared by cutting off the tops of acid‐cleaned PE bottles with a ceramic knife and fitting acid‐cleaned polypropylene mesh into the top (Figure S1 Supporting Information). A solid fraction (>500 µm) was separated by sieving the sludge through the mesh under gravity. The remaining suspension was deemed the liquid sludge phase. To obtain the UF, 3 mL of the liquid sludge were treated by centrifugation using ultrafiltration membranes (PALL centrifugal devices, 100 kDa pore size), which were precleaned with 2 mL of 1 m HCl.

A microwave digestion procedure was used to treat all samples. To this end, 250–270 mg samples of the three sludge phases were weighed into Teflon microwave (MW) digestion vessels. Subsequently, 9 mL distilled 14.5 m HNO_3_ and 1 mL of 30% Suprapur H_2_O_2_ (VWR UK) were added to the vessels and left overnight for predigestion. The microwave vessels were then transferred to an Ethos EZ microwave system (Analytix Ltd) (1200 W) where digestion was achieved by heating the samples to 200 °C over 15 min and maintaining at this temperature for a further 15 min.

Following cooling, the solutions were transferred to 15 mL Teflon vials, evaporated to dryness, and redissolved in 1 mL of 1 m HCl. The 1 m HCl sample solutions were then processed through a one‐step anion exchange column chemistry procedure, which provides high‐purity Zn fractions at high yields, as outlined in previous studies.^[^ ^35^, ^36^, ^37^ ^]^

### Mass Spectrometry and Data Reduction

The isotopic analyses were conducted in the MAGIC Laboratories with a Nu Instruments Nu Plasma HR MC‐ICP‐MS and a Nu Instruments DSN desolvating sample introduction system. The measurements and subsequent data reduction employed established procedures so only a brief summary is provided here.^[^ ^19^, ^36^, ^37^ ^]^ The purified Zn fractions of the samples were dried down and redissolved in an appropriate volume of 0.1 m HNO_3_ to produce run solutions for mass spectrometry with total Zn concentrations of ≈40 to 70 ng mL^−1^. Finally, all run solutions were doped with Cu to a concentration of 50 ng mL^−1^.

The isotopic analyses encompassed monitoring the ion beam intensities of the four main Zn isotopes (^64^Zn, ^66^Zn, ^67^Zn, ^68^Zn), ^63^Cu, and ^65^Cu, as well as ^62^Ni, to correct for the isobaric interference of ^64^Ni on ^64^Zn, using Faraday cups. The measured Zn isotope ratios were corrected “online” for instrumental mass bias relative to the ^65^Cu/^63^Cu isotope ratio. For quality control, the sample analyses were interspersed between regular measurements of 50 ng mL^−1^ solutions of the in‐house “London Zn” reference material. As expected, the solid, liquid, and UF samples from the control experiment to which no ^64^Zn‐en and ^68^Zn‐en were added (Table 1) revealed Zn isotope compositions that were identical within better than 0.2% to the London Zn standard. The same result was obtained for an aliquot of the London Zn solution that was processed through the column chemistry; this analysis also confirmed that the column chemistry procedure consistently achieved yields of better than 92%. A full procedural blank was processed alongside samples, and this confirmed that the Zn contamination from sample preparation was insignificant, at ≤1% of the indigenous Zn present in the samples.

Comprehensive data reduction was carried out “offline” following completion of the analyses.^[^ ^19^ ^]^ In detail, the molar fractions of Zn‐nat, ^64^Zn‐en, and ^68^Zn‐en present in the samples were obtained by forward‐modeling using the solver add‐in of Microsoft Excel, by minimizing the differences between the ^68^Zn/^64^Zn, ^64^Zn/^66^Zn, and ^68^Zn/^66^Zn ratios that were measured for the samples (following mass bias correction) and that were be predicted by mixing calculations. Furthermore, the ^66^Zn concentrations of the sample solutions were determined, relative to a calibration curve, which was defined by analyses of standard solutions with variable Zn concentrations and using the added Cu as internal standard. Finally, the mass amounts and concentrations of the three Zn species in the samples were calculated based on i) the known Zn isotope compositions of the three “endmember” Zn species (Zn‐nat, ^64^Zn‐en, ^68^Zn‐en); ii) the measured Zn isotope compositions of the samples; iii) the initially determined ^66^Zn concentrations of the samples; and iv) the sample masses and dilution factors employed to prepare the run solutions. Based on repeat analyses of quality control samples, the Zn mass amounts and concentrations had a repeatability (± one standard deviation for multiple runs of a single sample) of better than 1% (primarily limited by variable instrumental sensitivity) and a bias of less than 8%, primarily limited by the slightly variable yield of the sample preparation procedure. As instrumental sensitivity and yields have no impact on the measured molar fractions of Zn‐nat, ^64^Zn‐en, and ^68^Zn‐en, these parameters had a bias and repeatability (± one standard deviation) of better than 0.5%.

## Conflict of Interest

The authors declare no conflict of interest.

## Supporting information

Supporting Information

## Data Availability

The data that support the findings of this study are available from the corresponding author upon reasonable request.
