# Supplementary material for: ZnO Nanomaterials and Ionic Zn Partition within Wastewater Sludge Investigated by Isotopic Labeling
Source: Glob Chall. 2022 Jan 5;6(3):2100091. doi: 10.1002/gch2.202100091 (PMC8902288; doi:10.1002/gch2.202100091)
Supplement: Supplementary file 1 — Supporting Information [file GCH2-6-2100091-s001.pdf]

Global Challenges
Open Access

Supporting Information

for *Global Challenges,* DOI: 10.1002/gch2.202100091

ZnO Nanomaterials and Ionic Zn Partition within Wastewater Sludge Investigated by Isotopic Labeling

*Miguel A. Gomez-Gonzalez, Mark Rehkämper, Zexiang Han, Mary P. Ryan, Adam Laycock, and Alexandra E. Porter**

Supporting Information

ZnO Nanomaterials and Ionic Zn Partition Within Wastewater Sludge Investigated by Isotopic Labeling

*Miguel A. Gomez-Gonzalez, Mark Rehkämper, Zexiang Han, Mary P. Ryan, Adam Laycock, Alexandra E. Porter**

Table S1. Summary of past literature on the behavior of ZnO nanomaterials in wastewater media, compared to this work.

Wastewater media and their characteristics	ZnO ENM characteristics [a]	Spiking concentration [$\mu g\ g^{-1}$]	Incubation period	References
Primary sludge, Anglian Water, UK (pH = 5.2)	NPs: 7.6 nm in diameter	10.2	4 h	This work
Activated sludge from California, USA	Three types of NPs<br>a. 100 nm in diameter<br>b. 300 nm in diameter<br>c. 50–300 nm	2, 10	7 days	Smeraldi *et al.*[1]
Simulated influent (pH = 7)	Highly heterogeneous in size (10–130 nm) and shape	5, 10, 20	21 days	Chaüque *et al.*[2]
Real sewage from South Australia, Australia	NPs: 22.9 nm in diameter	700	3 days	Brunetti *et al.*[3]
Simulated sludge (humic acid; pH = 4.5)	Nanorods: 139 nm in diameter and ~5.3 in aspect ratio	1000	3 h	Gomez-Gonzalez *et al.*[4]
A mixture of primary and activated sludge from South Australia, Australia (pH = 7.2)	Three types of NPs:<br>a, b. 30–40 nm in diameter<br>c. Co-doped ZnO with hydrodynamic size d = 35 nm	1000	10 days	Lombi *et al.*[5]

[a)] Unless otherwise specified, NP size/diameter refers to bare size as measured by transmission or scanning electron microscopy.

Standard operating procedure for the synthesis of ^{68}ZnO nanoparticles

1) 35 mL acetic acid are heated to 89°C under stirring and with use of a reflux condenser.

2) 390 mg ^{68}Zn metal are added to the acetic acid and the reaction is left to run for 72 hours.

3) After cooling, the ^{68}Zn acetate present in the bottom of the vessel is removed, transferred to a petri dish and dried in an oven at 60°C overnight.

4) 100 mg ^{68}Zn acetate is dispersed in 50 mL diethylene glycol (DEG) by stirring for 15 min. The glass beaker with this mixture is then placed in oven at 60°C for 72 hours.

5) Silicone oil is heated in a metal or glass bath to 177°C. Once a stable temperature has been reached, the glass beaker with the DEG-^{68}Zn acetate mixture (covered with a foil lid with pierced holes) is placed in the silicone oil bath.

6) When the DEG-^{68}Zn acetate mixture reaches 170°C, 100 µL ultrapure water is added to hydrolyze the ^{68}Zn acetate and the mixture is then stirred at 350 rpm.

7) The DEG starts to turn yellow after about 30 minutes; at this point the glass beaker is removed from the silicone oil bath and left to cool.

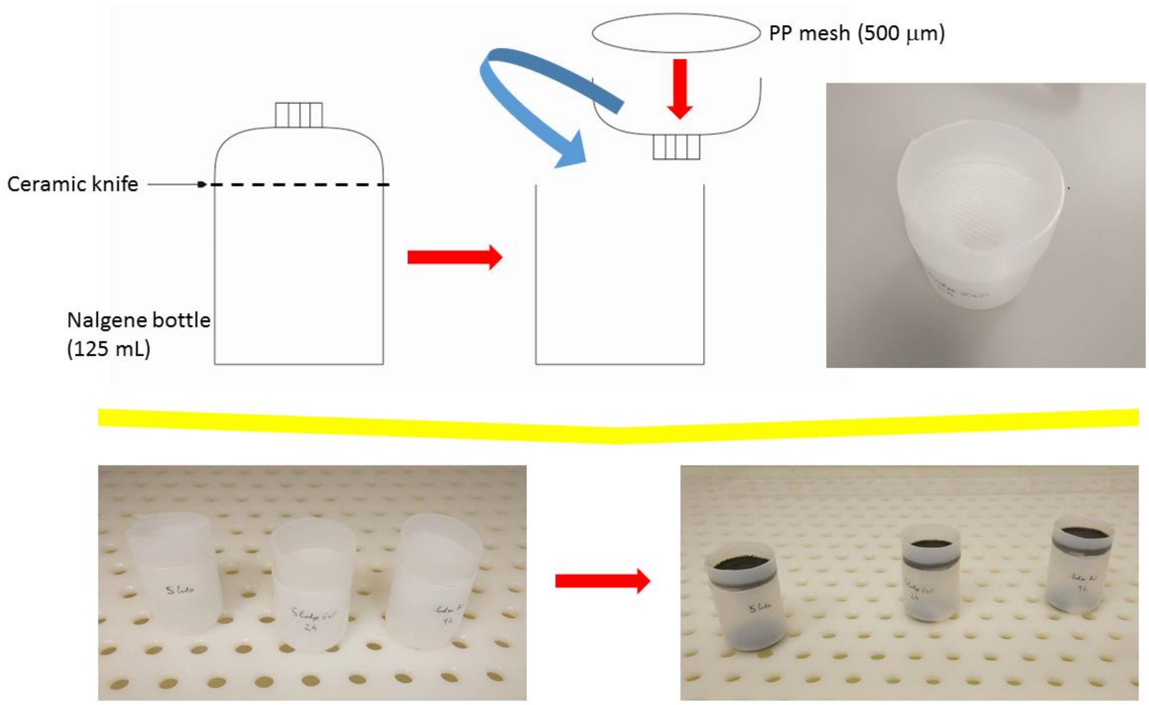

Figure S1. Procedure followed for sieving the sludge samples and general view.

Table S2. Summary of the primary sludge samples studied with and without enriched Zn addition.

	Samples	Added species	Incubation time	Characteristics
**1**	Solid	-	-	Solid sludge (>500 µm) with no added ZnO NPs
**2**	Solid	^{68}ZnO $^{64}ZnCl_2$	30 min	Solid sludge (>500 µm) with labeled Zn added, and incubated during 30 min under orbital shaking
**3**	Solid	^{68}ZnO $^{64}ZnCl_2$	4 h	Solid sludge (>500 µm) with labeled Zn added, and incubated during 4 hours under orbital shaking
**4**	Liquid	-	-	Liquid sludge with no added ZnO NPs
**5**	Liquid	^{68}ZnO $^{64}ZnCl_2$	30 min	Liquid sludge with labeled Zn added, and incubated during 30 min under orbital shaking
**6**	Liquid	^{68}ZnO $^{64}ZnCl_2$	4 h	Liquid sludge with labeled Zn added, and incubated during 4 hours under orbital shaking
**7**	UF fraction	-	-	Ultrafiltered fraction (< 2-3 nm) with no added ZnO NPs
**8**	UF fraction	^{68}ZnO $^{64}ZnCl_2$	30 min	Ultrafiltered fraction (< 2-3 nm) with labeled Zn added, incubated during 30 min under orbital shaking
**9**	UF fraction	^{68}ZnO $^{64}ZnCl_2$	4 h	Ultrafiltered fraction (< 2-3 nm) with labeled Zn added, incubated during 4 hours under orbital shaking

Table S3. Summary of the concentrations and masses of natural and the two enriched Zn species measured by MC-ICP-MS for the experimental systems.

	Concentration of Zn species [μg g^{-1}] [a)]			Mass of Zn species μg for 10 g sludge sample [b)]		
**Sample**	**Natural Zn**	**^{68}Zn-en**	**^{64}Zn-en**	**Natural Zn**	**^{68}Zn-en**	**^{64}Zn-en**
Solid (no Zn added)	45.5	0.00	0.01	18.7	0.00	0.00
Solid 30 min	50.9	30.5	14.9	21.0	12.6	6.14
Solid 4 h	44.6	24.1	11.4	18.4	9.91	4.69
Liquid (no Zn added)	6.26	0.0	0.0	60.1	0.00	0.00
Liquid 30 min	2.02	2.31	1.15	19.4	22.2	11.0
Liquid 4 h	2.96	2.65	1.25	28.4	25.4	12.0
UF (no Zn added)	0.89	0.00	0.00	8.52	0.00	0.00
UF 30 min	1.35	2.67	1.33	12.9	25.6	12.7
UF 4 h	1.90	3.01	1.43	18.2	28.9	13.7

[a)] The unit μg g^{-1} denotes μg of detected Zn species per gram of a given phase (solid, liquid, or UF = ultrafiltrate) of the primary sludge. [b)] Mass of Zn present in the three phases of the experimental system with 10 g of sludge; 10 g of sludge encompass 0.412 g solid and 9.588 g liquid. The Zn mass amounts and concentrations have a bias of less than 8%.

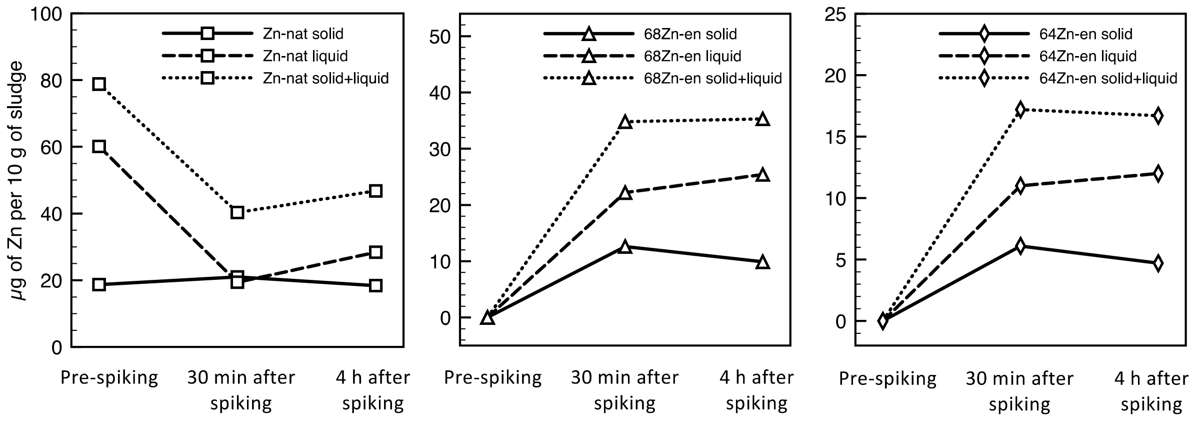

Figure S2. Zn mass budget, showing re-equilibration of Zn species within sludge samples over time. Slight variations observed from 30 min to 4 h reflect slow re-equilibration of Zn within the experimental system, including the surfaces of the polyethylene bottles (see text for details). The Zn mass amounts have a repeatability and bias of less than 1 and 8%, respectively.

Table S4. Calculated solid-liquid partition coefficients for the added ^{68}Zn-en and ^{64}Zn-en.

	**^{68}ZnO NPs**			**$^{64}ZnCl_2$ salt**		
	Concentration of ^{68}Zn in the solid [µg g^{-1}]	Concentration of ^{68}Zn in the liquid [µg g^{-1}]	Solid-liquid partition coefficient $D_{S/L}$	Concentration of ^{64}Zn in the solid [µg g^{-1}]	Concentration of ^{64}Zn in the liquid [µg g^{-1}]	Solid-liquid partition coefficient $D_{S/L}$
**30 min**	30.5	2.31	13.2	14.9	1.33	12.9
**4 h**	24.1	2.65	9.1	11.4	1.43	9.1

References

[1] J. Smeraldi, R. Ganesh, T. Hosseini, L. Khatib, B. H. Olson, D. Rosso, *Water Environ. Res.* **2017**, *89*, 880.

[2] E. F. C. Chaüque, J. N. Zvimba, J. C. Ngila, N. Musee, *Water SA* **2016**, *42*, 72.

[3] G. Brunetti, E. Donner, G. Laera, R. Sekine, K. G. Scheckel, M. Khaksar, K. Vasilev, G. De Mastro, E Lombi, *Water Res.* **2015**, *77*, 72.

[4] M. A. Gomez-Gonzalez, M. A. Koronfel, A. E. Goode, M. Al-Ejji, N. Voulvoulis, J. E. Parker, P. D. Quinn, T. B. Scott, F. Xie, M. L. Yallop, A. E. Porter, M. P. Ryan, *ACS Nano* **2019**, *13*, 11049.

[5] E. Lombi, E. Donner, E. Tavakkoli, T. W. Turney, R. Naidu, B. W. Miller, K. G. Scheckel, *Environ. Sci. Technol.* **2012**, *46*, 9089.

[6] M. A. Gomez-Gonzalez, M. A. Koronfel, H. Pullin, J. E. Parker, P. D. Quinn, M. D. Inverno, T. B. Scott, F. Xie, N. Voulvoulis, M. L. Yallop, M. P. Ryan, A. E. Porter, *Adv. Sustain. Syst.* **2021**, *5*, 2100023.

[7] A. L. Fabricius, L. Duester, B. Meermann, T. A. Ternes, *Anal. Bioanal. Chem.* **2014**, *406*, 467.
